# TransAnal Total Mesorectal Excision (TaTME) in Peru: Case series

**DOI:** 10.1016/j.ijscr.2020.09.204

**Published:** 2020-10-07

**Authors:** Andrés Guevara Jabiles, Francisco Berrospi Espinoza, Iván Klever Chávez Passiuri, Eduardo Payet Meza, Carlos Emilio Luque-Vásquez, Eloy Ruiz Figueroa

**Affiliations:** Department of Abdominal Surgery, INEN, Angamos Este 2520 Ave, Surquillo, 15038 Lima, Peru

**Keywords:** TaTME, Total mesorectal excision, Rectal cancer surgery, Peru, Case series

## Abstract

**Introduction:**

Describe and demonstrate the feasibility and safety of TaTME in short term outcomes in the Instituto Nacional de Enfermedades Neoplásicas (INEN) in Peru.

**Materials and methods:**

Case series with retrospective and prospective data collection of patients with middle and inferior rectal cancer who underwent TaTME between January 2015 and March 2020. Patients and tumor characteristics, operative details, postoperative complications and pathological results were analyzed.

**Results:**

Nineteen patients were included. The median age was 56 years old (range 40–69). Ten were female. The median distance from the anal verge was 4 cm (range 3–6) and 17 cases were located in the inferior rectum. Eleven patients with clinical stage III. Thirteen (68.4%) patients received neoadjuvant treatment. There was no conversion to open surgery reported. Ten (52.6%) cases had intersphincteric resection and 18 (94.7%) had primary coloanal anastomosis, 13 (72.2%) of them with hand-sewn. All patients had a diversion with ileostomy. The median operative time was 330 min (range 270–480). Median postoperative hospital stay of 5 days (range 3–18). The overall rate of postoperative complication was 21.1%, two cases (10.5%) had anastomotic leakage and mortality was present in one (5.3%) patient. 94.5% had an optimal TME specimen, only one case (5.3%) had positive circumferential resection margin and positive distal margin. The median tumor size in the specimen was 4 cm (range 2–11) and nine (47.4%) patients had ypT3 on pathology.

**Conclusion:**

TaTME is a safe and feasible technique with good pathological results.

## Highlights

• Transanal total mesorectum excision is feasible for mid and low rectal cancer.
• Good quality of the mesorectum specimen is obtain after TaTME surgery.
• TaTME with intersphincteric resection is a feasible option for selected cases of very low rectal cancer.
• Surgical complication rates after intersphincteric TaTME with hand-sewn coloanal anastomosis could be higher.

## 1 Introduction

The base of treatment for cancer of the middle and lower rectum is the low anterior resection with total mesorectal excision (TME) described by Heald et al. [[1], [2], [3]]. With the development of medical technology, minimally invasive surgery can replace open surgery in certain cases, and several randomized clinical studies showed that laparoscopic TME (lap TME) has advantages in the short term, and no inferiority in long term compared to open surgery [[4], [5], [6]]. However, the surgical approach in lap TME for tumors located in the middle and lower rectum continues to be a challenge for tumors located below peritoneal reflection. Trans anal Total Mesorectal Excision (TaTME) was first described in 2010 [7] as one of new surgical procedures including the surgical innovations of the last three decades for the approach to middle and lower rectal cancer: TME, minimally invasive surgery and natural orifice transluminal endoscopic surgery (NOTES) [8]. It was described as a solution to the known problems [9] since it promised a better quality of the total excision of the mesorectum, with low rate of positive circumfernetial resection margin (CRM) and low rate of positive distal margin (DM), and low conversion rate in patients with narrow pelvis, with benign prostatic hypertrophy (BPH) and obesity [1,10]. Large series and systematic reviews corroborate these affirmations and confirms superiority or equivalence in short and middle-term outcomes over the laparoscopic TME technique [2,11,12]. However, long-term oncological and functional results are still uncertain, but with promising results of non-inferiority [13,14].

The first TaTME procedure in Peru was performed in January 2015 at the Instituto Nacional de Enfermedades Neoplásicas (INEN) for the management of middle and low rectum pathology, below peritoneal reflection. Patients who required partial and/or total interesphincteric resection were included. This case series has been written according to the PROCESS guideline [15] and the aim is to audit our results. Demonstrate the feasibility of the procedure according to the resection parameters of an adequate surgical specimen and the safety of the procedure according to the number of complications, hospital stay and mortality.

## 2 Materials and methods

TaTME began in INEN Instituto Nacional de Enfermedades Neoplá of Peru since January 2015 for patients with benign neoplasia and middle and low rectum cancer candidates for low anterior resection or abdominoperineal resection with lesions located above the pectineal line without infiltration of the levator ani muscle nor of the anal sphincter. Data collection carried out retrospectively and prospectively until March 2020 in INEN. UIN number: researchregistry6040. Only cases with adenomatous polyp biopsy with high-grade dysplasia and rectal cancer were included. Patients with hereditary polyposis syndromes without rectal cancer were excluded for the final analysis. Locally advanced rectal cancer cases (cT3 and/or cN+) staged through Computed Tomography (CT) of the abdomen and pelvis and Magnetic Resonance Imaging (MRI) received concurrent neoadjuvant chemotherapy with Capecitabine 825 mg/mts2 concurrent to radiotherapy in doses of 50.4 cGys in 28 sessions. They had a clinical reevaluation to classify them according to their clinical response: incomplete (ICR), near complete (nCRC) and complete (CCR); according to the description by the Memorial Sloan Kettering regression scheme [16] between 8 and 12 weeks through digital rectal examination, proctoscopy, MRI of the pelvis and CT of the abdomen and pelvis. The tumor regression grade by magnetic resonance imaging (mrTRG) was used for reassessment after neoadjuvant treatment since 2018 [17]. Ethical approval was not needed.

### 2.1 TaTME surgical technique

All patients were informed and signed the surgical authorization. The TaTME involves resection of the rectum with total excision of the mesorectum using two approaches: 1) Laparoscopic: transabdominal and 2) Endoscopic: transanally. It began with a sequential approach; however, we currently carry out the simultaneous approach by two trained surgeons. The procedure starts abdominally through laparoscopic TME with high ligation of the inferior mesenteric vessels and routine mobilization of the splenic flexure. The trans anal approach is made by one very trained surgeon and starts simultaneously, with partial or total intersphincteric resection when the tumor is less than 1 cm from the anorectal junction on physical examination and MRI (type II rectal cancer according to Rullier classification) [18] and later the purse-string is made to close the rectal stump. Washing is perform with isodine solution at 1% and the disposable (GelPOINT Path Trans anal, Applied Medical, USA) or the reusable (D-Port, Karl Storz) platform is placed. The dissection of the mesorectal plane is completely done transanally until peritoneal reflection and the junction with the transabdominal approach. The surgical specimen extraction is made through the abdomen by a pfannenstiel incision of 4–6 cm with a wound protector retractor to prevent the risk of seeding malignant cells in the pelvis. The colon is prepared for hand-sewn or circular mechanical stapler coloanal anastomosis, according to the remainder of the length of the anal canal after trans anal rectotomy, preferably side-to-end. A protective loop ileostomy is usually done, and the placement of the pelvic drain is left to surgeon's discretion.

Demographic data, clinical characteristics of the tumor, the surgical procedure, complications and pathological anatomy results were collected. The surgical specimens were freshly processed for histopathological examination based on the method described by Phil Quirke et al. [19]. The quality of the mesorectum was classified as complete, almost complete or incomplete. Additional information on tumor-free circumferential resection margin (> 1 mm), distal margin, the number of resected nodes and the number of metastatic nodes was reported.

Complications were described. Intraoperative complications such as excessive bleeding, defined as ≥500 ml or bleeding with clinical repercussion on patient's hemodynamic status and the postoperative complications defined as complications presented within 30 days after surgery, classified according to Dindo-Clavien et al. [20] were described. Anastomotic leakage after low anterior resection of the rectum was classified as grade A, without clinical symptoms and normal laboratory tests, grade B, presenting clinical repercussion but only requires medical treatment, and grade C, requiring redo surgery [21]. The hospital stay was calculated from the operative day to the day of discharge. The enhanced recovery after surgery program was applied since 2019 (ERAS protocol).

### 2.2 Statistical analysis

Numerical data were determined with measures of central tendency, such as median and range; and categorical data, with counts and percentages.

## 3 Results

21 TaTME cases have been performed since 2015, seven cases until 2018 and 14 cases from January 2019 to March 2020. Two cases were excluded due to benign pathology (juvenile polyposis syndrome associated with BMPR1A gene pathogenic variant and another with serrated polyposis syndrome) leaving 19 cases (85.7%) with rectal malignant neoplasm, including the patient with low rectal neuroendocrine tumor were included in the final analysis. Table 1.

**Table 1:** Demographic and preoperative characteristics of 19 patients operated by TaTME for rectal cancer.

Characteristics	N (%)
Age (years)	56 (range 40–69)
Sex	
Male	9 (47.4%)
Female	10 (52.6%)
BMI (kg/m2)	26.2 (range 21–39)
Distance from the anal verge (cm)	4 (range 3–6)
Distance < 1 cm from anorectal junction	10 (52.6%)
Clinical tumor size (cm)	6 (range 2–11)
Location in rectum	
Inferior	17 (89.5%)
Middle	2 (10.5%)
Intraluminal tumor location	
Circumferential	5 (26.3%)
Antero lateral	6 (31.6%)
Postero lateral	3 (15.8%)
Anterior	3 (15.8%)
Posterior	2 (10.5%)
Polyp with high grade dysplasia	3 (15.8%)
CEA (ng/mL)	3.27 (range 1.09–398.8)
Clinical stage	
I	6 (31.6%)
II	1 (5.3%)
III	11 (57.9%)
IV	1 (5.3%)
Neoadjuvant CRT	13 (68.4%)

CRT: chemoradiotherapy.

The median follow up for clinical reassessment after neoadjuvant chemoradiotherapy (CRT) for the 13 patients was 9.6 weeks (range 5.4–21.4). Eight cases (61.5%) had ICR, four cases (30.8%) nCRC and only one patient (7.7%) with CCR. Seven patients had a post-treatment mrTRG scale measurement and only three had good response (mrTRG 1/2) to neoadjuvant therapy.

None of the patients operated by TaTME required conversion to open surgery. All patients had TaTME and laparoscopic approach, the first two cases had a sequential approach, however, the following 17 cases had a simultaneous approach (two surgical teams). The median operative time was 330 min (270–480). The clinical stage IV case due to one liver metastasis had TaTME surgery with laparoscopic S6 liver metastasectomy in a single surgery in 270 min.

**Fig. 1:**
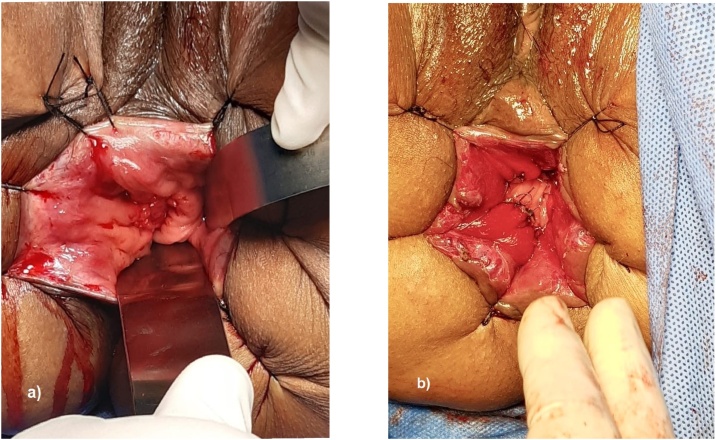
a) Residual inferior rectal cancer after neoadjuvant treatment. b) Hand-sewn coloanal anastomosis.

Ten patients (52.6%) had type II inferior rectal cancer according to the E. Rullier classification and required interesphincteric resection before placement of the trans anal platform. Eighteen patients (94.7%) had coloanal anastomosis with different techniques: 13 (72.2%) with hand-sewn, of which eight were side-to-end (Fig. 1), four were end-to-end and one case with a colonic J pouch. The remaining five cases had anastomosis with circular mechanical stapler, three patients with side-to-end technique and two with end-to-end technique. Since 2018, we prefer to use the side-to-end technique. Eighteen cases with primary anastomosis had protective ileostomy.

Two cases (10.5%) presented intraoperative complications, both due to excessive bleeding. There was no need for conversion to open surgery but blood transfusion was needed in the postoperative. No intestinal perforations or inadvertent vesicoureteric injuries were reported. Four (21.1%) presented postoperative complications within the first 30 days. One patient with prolonged ileus which remitted with medical treatment while the remaining three had major complications. One case with perianastomotic collection after discharge that required percutaneous drainage and parenteral nutrition, and two cases (10.5%) required surgical redo for anastomotic dehiscence, one of which did not require of Intensive Care Unit but was managed with parenteral nutrition with a hospital stay of 18 days. The other patient died on the third postoperative day from sepsis. Only one patient presented partial stenosis of the coloanal anastomosis treated with finger and endoscopic dilation between the second and fourth month after surgery. Table 2.

**Table 2:** Operative and postoperative characteristics of 19 patients operated by TaTME for rectal cancer.

Postoperative characteristics	N (%)
Operative time (min)	330 (270–480)
Intraoperative blood loss (ml)	150 (50–500)
Interesphincteric resection	10 (52.6%)
Coloanal anastomosis	18 (94.7%)
Hand-sewn	13 (72.2%)
Mechanical circular stapler	5 (27.8%)
Loop ileostomy	18 (94.7%)
Extraction of surgical specimen	
Abdominal	13 (68.4%)
Transanal	6 (31.6%)
Intraoperative complication	2 (10.5%)
Postoperative complication [CD grade III-V]	4 (21.1%) [3]
Postoperative hospital stay (days)	5 (3–18)

DC: Dindo-Clavien classification.

**Fig. 2:**
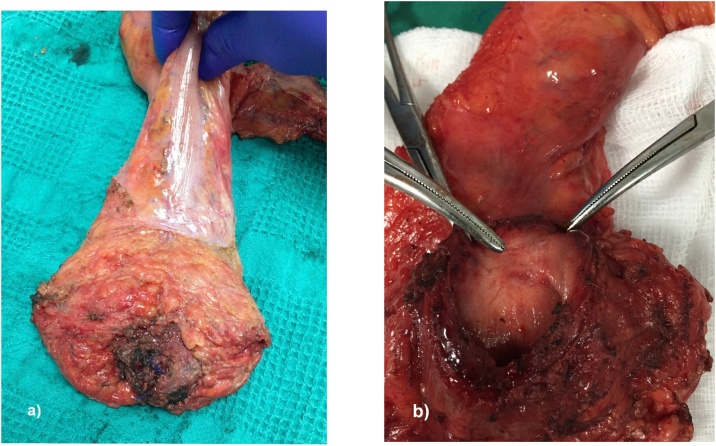
Surgical specimen after intersphincteric TaTME in a patient with ypT2 regrowth of inferior rectal cancer after neoadjuvant treatment.

The pathological results from our 19 cases showed that nine (47.4%) had pT3, despite the fact the majority received neoadjuvant therapy (Fig. 2). Two patients with a complete pathological response were classified as nCRC in the post-treatment clinical reassessment. Eighteen patients (94.7%) had tumor-free CRM (>1 mm), and tumor-free DM, only one case had positive DM and positive CRM and One patient (5.3%) had incomplete mesorectal quality specimen. Table 3.

**Table 3:** Pathological characteristics of 19 patients operated by TaTME for rectal cancer.

Pathological characteristics	N (%)
pT	
pT0	2 (15.8%)
pTis	4 (21.1%)
pT1	2 (10.5%)
pT2	2 (10.5%)
pT3	9 (47.4%)
pN	
pN0	15 (79%)
pN1	4 (21.1%)
Tumor size (cm)	4 (range 2–11)
Number of lymph nodes retrieved	23 (range 14–64)
Distal margin (mm)	10 (range 0–30)
Circumferential resection margin (mm)	4.5 (range 0–7)
Mesorectum quality specimen	
Complete	12 (63.2%)
Near complete	6 (31.6%)
Incomplete	1 (5.3%)

## 4 Discussion

TaTME surgery for middle and lower rectal cancer has demonstrated its roved effectiveness since its inception [10,22], and nowadays with a high number of cases worldwide, its safety has been proved [11,23]. In our experience, the TaTME technique began consolidating since January 2019, as the number of cases per year tripled. INEN is a tertiary referral cancer institute in Peru that presents the first and the highest number of cases carried out in the country at present. TaTME should only be performed in high-volume medical centers by experience surgeons, to keep the learning curve under strict supervision. This will avoid a negative impact on patients and improve short and long-term results [24,25].

The potential benefits of TaTME over Lap TME were based on the results of the comparative studies between laparoscopic and open surgery [4,5] given its high conversion rate to open surgery. However, the conversion rate of TaTME surgery is less than 1% reported in various studies [11,23] as well as in our series where none of the 19 cases required conversion. This shows the feasibility of performing surgery through a minimally invasive approach. Subsequently, Ma et al. [12] showed in a systematic review that the conversion rate in TaTME surgery is much lower than in Lap TME surgery, due to the greater visibility of the pelvis and comfort, especially in patients with unfavorable characteristics.

Another advantage of the TaTME over the Lap TME is the lower intraoperative bleeding and the shorter operative time due to the use of two surgical teams in a simultaneous approach, abdominal and trans anal approach [1,12]. The median intraoperative bleeding in our case series was 150 ml (range 50–500 ml), similar to the first 20 cases reported by Atallah S et al. [8], however, in series with more than 50 cases and in multicenter studies, the bleeding volume is less than 80 ml [23,26]. The median operating time in our study was 330 min, similar to series with 22 cases in Russia [27], however, longer than Lacy A et al. [11] and Perdawood SK et al. reports [23]. Difference could be related to the learning curve and the number of cases carried out by each institution as the Danish experience described that the time of the trans anal approach was reduced in the last 75 cases of TaTME. Likewise, 72% (13/18) of the patients in our series required hand-sewn suturing for anastomosis and intersphincteric resection, unlike 28.6% reported by Lacy A et al., which could increase the operating time given the complexity of the technique.

The enhanced recovery after surgery protocol (ERAS) for patients with colorectal elective surgery began in INEN on January 2019 and 12 patients (63.2%) with TaTME surgery were included in the program. The median hospital stay was 5 days, similar to other studies ranging from 4.5–7 days [8,22]. However, the multicenter study in China [26] reported up to 13 days, because they started late diet tolerance and because the patients did not have a protective derivative loop ileostomy compared to the other studies described [11].

Four patients (21.1%) presented post-operative complications and the majority were major complications (15.8%). Two cases (10.5%) with leakage, both required redo surgery. Other studies report an overall complication rate of up to 35%, the majority minor complications, leakage average rate of 8.6% and only 6.6% who required redo surgery [11,26]. This difference could be related to the location in the lower rectum of most of our patients, the shorter distance between the anal margin and the tumor, the hand-sewn anastomosis and the higher number of cases with neoadjuvant treatment (68.4% vs. 27.5%) in comparison to other series [11]. The use of neoadjuvant chemoradiotherapy, the shorter distance to resection and hand-sewn anastomosis are factors that increase the risk of dehiscence after a previous low resection by TaTME [28,29], This explains this the slight increase in leakage rate in our case series compared to the mentioned studies.

The TaTME technique reduces the complexity of the deep pelvis approach and improves the quality of the surgical specimen (tumor-free distal and circumferential resection margin and good quality of complete/almost complete mesorectum), as some comparative studies between Lap TME and open surgery failed to show this reporting up to 12% of positive CRM [30,31]. Eighteen patients (94.7%) in our study had an adequate mesorectum quality (complete in 63.2% and almost complete in 31.6%), better than the Danish study however; the study by Lacy A et al. [11] and the multicenter study from China [26] reported a percentage less than 1.4%.

The median CRM and DM in our study was 4.5 mm (range 0–7) and 10 mm (range 0–30) respectively, with a positive CRM and positive DM of 5.3%. Similar results are reported by other authors between 6.4% and 2.3% [11,23,26], however, the average DM towards the tumor varies between 19 mm and 28 mm compared to the 10 mm in our series. The majority (53%) of our cases had cancer of the lower rectum located less than 1 cm from the anorectal junction requiring intersphincteric resection (ten cases).

Finally, the median tumor size in the surgical specimen in our series was greater (4.0 vs 3.3 cm) compared to other studies [26,32]. In addition to the size of the tumors reported and the use of neoadjuvant treatment in most of the cases, tumors of the middle and lower rectum managed in our institution are bigger and more advanced than in other latitudes, which technically, are more challenging for surgeons.

## 5 Conclusion

The study demonstrates that TaTME technique for middle and low rectal cancer is feasible in a high-volume institution in Peru with specially trained surgeons to obtain acceptable short-term results. Further follow up is needed for long term and functional outcomes and a prospective study is recommended for comparison with laparoscopic TME.

## Declaration of Competing Interest

No conflicts of interest.

## Funding

No funding for research.

## Ethical approval

The study is exempt from ethical approval in our institution.

## Consent

Written informed consent was obtained from the patients for publication of this case series. There are figures and pictures of the patients protected by anonymity.

## Author contribution

Iván Klever Chávez Passiuri: Formal analysis.

Eduardo Payet Meza: Formal analysis and Supervision.

Carlos Emilio Luque-Vásquez: Visualization.

Eloy Ruiz Figueroa: Validation.

## Registration of research studies

1. Name of the registry: researchregistry6040
2. Unique identifying number or registration ID: 6040
3. Hyperlink to your specific registration (must be publicly accessible and will be checked): https://www.researchregistry.com/browse-the-registry#home/

## Guarantor

Andres Guevara Jabiles.

## Provenance and peer review

Not commissioned, externally peer-reviewed.
